# Specialists and Specialties

**Published:** 1897-12

**Authors:** 


### SPECIALISTS AND SPECIALTIES.

A medical specialist differs widely from the poet, who, we are told, is "born, not made." The medical specialist is made, not born. No specialist has ever yet been born full-fledged from a medical college, though occasionally a young man imagines that such a miracle has been performed. The fledgling who supposes that a medical diploma and a post-graduate course can make a specialist deceives himself, and endangers the safety of the community. Such specialists have done untold harm to the profession. The eye, the ear, the skin, are not simple appendages, each performing its duties independent of the rest, to be treated without reference to the other organs and the condition of the body at large. The successful specialist must also be a competent general physician. In no department of practice is this more true than in pediatrics. No man can ever become a successful practitioner among children who has not a broad and thorough knowledge of general medicine.—*Arch. of Pediatrics.*

